# Dual mediating effects of social support and fertility stress on mindfulness and fertility quality of life in infertile men: A case-control study

**DOI:** 10.3389/fpsyg.2023.1138282

**Published:** 2023-03-13

**Authors:** Maierhaba Abulizi, Hua Xu, Alapate Abuduguli, Wanzhu Zhao, Lijuan He, Chen Zhang

**Affiliations:** ^1^School of Public Health, Xinjiang Medical University, Urumqi, Xinjiang, China; ^2^The First Affiliated Hospital of Xinjiang Medical University Reproductive and Fertility Centre, Urumqi, Xinjiang, China; ^3^Postdoctoral Station, School of Pharmacy, Xinjiang Medical University, Urumqi, Xinjiang, China

**Keywords:** infertility, fertility quality of life, social support, fertility stress, mindfulness

## Abstract

**Background:**

Infertility is one of the three major public health problems in the world, bringing immense physical and psychological damage to men and affecting the quality of men’s fertility life. Thus, the purpose of this study was to analyze the status of social support, fertility stress, mindfulness, and fertility quality of life in infertile men, and to explore the dual mediating effects of social support and fertility stress on mindfulness and fertility quality.

**Methods:**

A case–control group study was conducted, with 246 men in the case group and 149 in the control group. The Social Support Scale, Fertility Stress Scale, Mindfulness Scale, and Fertility Quality of Life Scale were used to establish a structural equation model using Mplus 8.3 to explore social support and fertility stress. Pathway relationships were drawn between mindfulness and fertility quality of life in infertile men.

**Results:**

There were significant differences between infertile and healthy men in each dimension of the core module of fertility quality of life, in the total score of the treatment module, in the total score of social support, in subjective and objective support, and in the total score of fertility stress, social pressure, sexual pressure, marital relationship, and childless pressure (*p* < 0.05 in each case). Further, the fertility quality of life in infertile men was positively correlated with mindfulness and social support, and negatively correlated with fertility stress (*p* < 0.05); mindfulness could directly affect the core and treatment modules of fertility life quality, and indirectly affect the core of fertility life quality through social support (mediation effect accounted for 19.0%), while the treatment module (mediation effect accounted for 13.7%), and the core module indirectly affected fertility life quality through fertility stress (mediation effect accounted for 16.8%).

**Conclusion:**

The fertility quality of life of infertile men is not optimistic. Mindfulness-related interventions and programs can improve their fertility quality of life.

## Introduction

According to the World Health Organization, male infertility is defined as the inability of women to conceive due to reasons pertaining to men, under the conditions of them sharing living arrangements for more than one year, having a normal sexual life, and not taking contraceptive measures during this period (Massarotti et al., 2019). The worldwide proportion of infertile couples is 15%, including male, female, and unknown reasons (Ried and Alfred, 2013). Previous studies have shown that there are many causes of male infertility, including genitourinary tract infections, chromosomal abnormalities, unhealthy lifestyles, and changes in fertility hormones (Wang et al., 2020). The number of male patients suffering from infertility is increasing daily, which not only affects their physical and mental health, but also deteriorates family relationships and increases the pressure on both spouses. Sex is not just about the fertility function; it can serve as a way of giving and receiving pleasure, maintaining intimacy between couples, and making a significant contribution to a person’s quality of life (Wang et al., 2017).

The concept of “quality of life” is defined by the World Health Organization as “a person’s perception of the culture and value system in which they live in life, and their relationship to their goals, expectations, standards and concerns” (Warchol-Biedermann, 2021). The fertility quality of life refers to the individual’s perception of their place in life within the fertility and treatment environment that men are exposed to Jing et al. (2020). Quality of life is an important part of the survival status of infertile patients, and is also one of the hot areas of concern for fertility medicine researchers (Ca and Cristiana, 2013). Men with primary infertility scored significantly lower in the emotional, physical, and social domains, indicating a poorer quality of life. For example, the quality of life of Japanese men is significantly higher than that of Turkish men. This may have an important correlation with the improvements in Japan’s infertility treatment system and social subsidies, while in Turkey, Iran, and other Middle Eastern countries, the lower level of quality of life of male infertility patients might be closely related to their traditional culture (Ahmadi et al., 2011).

Mindfulness is often defined as “a deliberate, non-judgmental awareness of the present moment” that arises through “purposeful, non-judgmental attention to the present moment” (Li J. et al., 2019). Short et al. considered that individuals with high dispositional mindfulness are able to regulate behavior and adapt to everyday life, resulting in stronger positive effects and lower intensity negative effects (Short et al., 2016). The findings confirmed that mindfulness is positively associated with a better fertility quality of life through self-regulation. Parto et al. showed that mindful behaviors and practices can help individuals reduce emotional reactivity, and that infertile men with high mindfulness act in a way that aligns with their needs and fertility values, thereby increasing tolerance to infertility treatment sex, and satisfaction with medical staff and services (Parto and Besharat, 2011).

Studies found that social support enhances mechanisms of stress, has positive effects on health (Asazawa et al., 2019), and can ease an individual’s stress-related emotional burden (Steuber and High, 2015). Further, infertile patients often perceive themselves as craving support from social network members (High and Steuber, 2014). Most studies have shown that social support is an important factor affecting the quality of life of male infertility patients (Schaller et al., 2016; Namdar et al., 2017; Maroufizadeh et al., 2018; Bhamani et al., 2020). With full support from family, society, hospital, and partners, male infertility patients will be more active in the treatment process and overcome their inner pain. However, they are reluctant to mention their infertility status to their partners, family members, colleagues, or in their social network, and even more reluctant to mention their feelings and worries to medical workers (Goker et al., 2017). Therefore, helping male infertility patients to find and benefit from a social support system can effectively improve their mood, while good social support can alleviate and improve their quality of life.

Fertility stress refers to the stress from social networks, marital relationships, as well as that related to physical and mental health due to infertility (Li Y. et al., 2019). Multiple studies have confirmed that fertility-related stress affect the quality of life of infertile patients, calling for more attention to this issue (Casu et al., 2018; Dehghan et al., 2020). Negative emotions, such as high levels of distress, guilt, sadness, and frustration associated with infertility impair the patient’s quality of life (Ried and Alfred, 2013). In addition to the stress of infertility itself, its diagnosis and treatment are often accompanied by significant stress. Infertility-related stress is one of the important predictors of mental health in infertile men, but little is known about the underlying mechanisms between these constructs, especially in developing countries (e.g., mainland China; Li X. et al., 2019).

Therefore, based on the aforementioned research, this study maintains that infertile men have lower fertility quality of life, lower social support, higher fertility stress, and lower levels of mindfulness. This study proposes that social support and fertility-related stress in infertile men could act as mediators between positive mindfulness and fertility quality of life, influencing the direct effect of positive mindfulness levels on both dimensions of quality of fertility life. Exploring the potential mechanisms between positive mindfulness and fertility quality of life in infertile men provides new ideas for clinical research that could help improve the fertility quality of life in infertile men by improving social support and fertility stress.

## Materials and methods

### Participants

The aim of this study was to examine the correlation between social support, fertility-related stress, positive mindset, and quality of life in fertility. For this purpose, men (*n* = 246) with infertility who visited the outpatient clinic of the Fertility and Infertility Centre of the First Affiliated Hospital of Xinjiang Medical University, China, from December 2020 to September 2022 were recruited as a case group and 149 healthy men who underwent health check-ups during the same period as a control group.

Inclusion criteria: ① Men of childbearing age who have normal sexual intercourse and are not using contraception, and whose wives have failed to conceive spontaneously within 1 year due to male factors, and whose women have regular menstruation and no significant abnormalities in gynecological fertility-related examinations. ② Abnormal semen routine examinations (Fifth edition of World Health tissue semen analysis standards). ③ Participation in the study was voluntary and informed consent was obtained from each person prior to conducting their assessment.

Excluded criteria: ① Patients with a major medical condition (tumor, serious mental illness, and serious chronic disease). ② A proven psychological disorder, a history of drug or substance use, alcohol addiction, experience in routine mind–body practice, psychological intervention, or who had used antipsychotic medication in the past 6 months. ③ Had difficulties in understanding and completing the questionnaire.

### Measures

#### Causes of infertility

The top four factors contributing to male infertility were, in order, abnormal semen, sexual dysfunction, prostatitis, and varicocele. All the subjects included in this study were men with abnormal semen quality leading to infertility, which may be accompanied by sexual dysfunction, prostatitis, and varicocele.

#### General characteristics questionnaire

The main contents of the questionnaire included items on participants’ age, ethnicity, place of residence, education level, weight, height, fertility history, past disease history, monthly family income, occupation, smoking history, drinking history, exercise time, sleep time, medical insurance classification, and environmental exposure history.

#### Mindful attention awareness scale

The Mindful Attention and Awareness Scale is a scale developed by Ryan and Brown in 2003 to measure mindfulness, which is individuals’ retention of present experiences in their daily lives—a general tendency to notice and be aware. The scale comprises 15 questions, which are rated on a 6-point Likert scale (from 1 = *almost always*, to 6 = *almost never*). Participants rated how often they experienced states such as autopilot thinking or preoccupation. The minimum and maximum scores on this scale are 15 and 90 points, respectively. The higher the score, the higher the level of mindfulness.

#### Social support rating scale

The Social Support Rating Scale includes three dimensions: objective support, subjective support, and support utilization. The higher the score, the better the social support. Questions 1–4 and 8–10 are single-choice questions; participants indicated their responses on a scale from 1 to 4, which correspond to scores 1–4, respectively. Question 5 has four sub-questions: A, B, C, and D; participants indicated their responses on a scale from 1 to 4, which correspond to scores 1–4, respectively. Questions 6 and 7 are multiple-choice questions. The responses to these questions were dichotomized into 0 = “no source” and 1 = the remaining choices (Spouse, other family members, relatives, colleagues, workplace, official or semi-official organizations such as parties and unions, and unofficial organizations such as social groups, other, and empty).

#### Fertility problem inventory

The fertility-related stress of infertile couples was measured using the Fertility Problem Inventory scale, which was specially designed by CR Newton in 1999 in Canada to evaluate fertility-related stress in infertile patients. The score and each subscale have high reliability and validity. The scale has a total of 46 items on 5 dimensions, including social pressure (10 items), sexual pressure (8 items), marital relationship (10 items), demand for parenthood (10 items), and rejection of a childless lifestyle (8 items). A Likert 6-point scale was used to calculate the score (from 1 = *completely disagree* to 6 = *completely agree*), the total score ranges from 46 to 276 points—the higher the score, the higher the level of fertility stress.

#### Fertility quality of life

The European Society for Human Reproduction and Embryology and the American Society for Fertility Medicine have jointly created the Fertility Quality of Life (FertiQoL) indicator with Merck KGaA Darmstadt, Geneva, Switzerland (Merck KGaA Darmstadt, a subsidiary of Merck, Germany). The overall goal of the FertiQoL project was to develop an international instrument to measure the quality of life of men and women with fertility problems. Its second purpose was to assess the psychometric properties of the tool. The scale consists of 2 items and 2 modules (core module and optional treatment module) of subjective overall quality of life and subjective overall health status, with a total of 36 items. The core module contains 24 items on 4 dimensions (including emotional responses, physical and mental relationships, marital relationships, and social relationships), with 6 items for each dimension; the optional treatment module contains 10 items, including the treatment environment (6 items) and tolerance (4 items) dimensions. Each item is scored from 0 to 4 points. By converting the scores of the total scale and subscales, the total score ranges from 0 to 100 points. The higher the score, the better the quality of life.

### Data analysis

The statistical analysis was performed in SPSS 26.0, and the model construction was performed using Mplus 8.3 software. First, the quantitative data were expressed as means, standard deviations, and interquartile ranges. R language was used to analyze the relationship between variables. The maximum likelihood estimation method was used to construct the model in Mplus 8.3 to explore the fit between the actual data and the theoretical model, as well as the direct and indirect influence paths and effect sizes. The Bootstrap program (repeated 5,000 times) was used to test whether the chain mediation effect was significant. The 95% confidence interval was used as the basis for judgment. If the interval did not contain 0, the mediation effect was significant, with the significance level at 0.05.

## Results

### Baseline data of the two groups of participants

The case group and the control group included 246 and 149 participants, respectively. The baseline data of the participants in the two groups are shown in Table 1.

**Table 1:** Baseline data of the two groups of subjects.

Variable	Case *n*(%)	Control *n*(%)
**Age(years)**		
≤32	139(56.5)	97(65.1)
>32	107(43.5)	52(34.9)
**Nationality**		
Han nationality	199(80.9)	130(87.2)
Minority	47(19.1)	19(12.8)
**Place of residence**		
City	207(84.1)	127(85.2)
Rural	39(15.9)	22(14.8)
**Education level**		
Junior high school and below	31(12.6)	10(6.7)
High school/secondary school	52(21.1)	22(14.8)
College/undergraduate	151(61.4)	110(73.8)
Master degree and above	12(4.9)	7(4.7)
**Monthly household income (yuan)**		
<4,000	45(18.3)	17(11.4)
4,000~6,999	106(43.1)	66(44.3)
7,000~9,999	55(22.4)	33(22.1)
>10,000	40(16.3)	33(22.1)
**Fertility history**		
None	234(95.1)	126(84.6)
Have (≥1)	12(4.9)	23(15.4)
**Body mass index**		
<18.50	24(9.8)	14(9.4)
18.50~24	137(55.7)	88(59.1)
24~28	66(26.8)	36(24.2)
>28	19(7.7)	11(7.4)
**Past medical history**		
Have	36(14.6)	16(10.7)
None	210(85.4)	133(89.3)
**Medicare reimbursement**		
Have	104(42.3)	73(49.0)
None	142(57.7)	76(51.0)
**Smoking**		
Yes	139(56.5)	90(60.4)
No	107(43.5)	59(39.6)
**Drinking**		
Yes	232(94.3)	142(95.3)
No	14(5.7)	7(4.7)
**Exercise**		
Never exercise or exercise occasionally	128(52.0)	75(50.3)
1–2 times/week	84(34.1)	51(34.2)
3–4 times/week	25(10.2)	16(10.7)
≥5 times/week	9(3.7)	7(4.7)
**Sleep**		
<6 h	40(16.3)	19(12.8)
≥6 h	206(83.7)	130(87.2)
**Exposure to harmful environment**		
Yes	51(20.7)	34(22.8)
No	195(79.3)	115(77.2)

### Both groups’ scores on each scale

As shown in Table 2, the control group obtained significantly higher total scores and scores on each dimension of fertility quality of life and social support compared to the case group. The differences between core fertility, physical and psychological health, emotional reactions, social relationships, marital relationships, and fertility treatment were significant (*p* < 0.05). The case group obtained significantly higher total scores and scores on each dimension of fertility stress and positive thinking than the control group. The differences between fertility stress, social stress, sexual pressure, conjugal relations, and parental role needs were significant (*p* < 0.05).

**Table 2:** Scores of each scale in the two groups.

	Item	Case	Control	*t*	*P*
FertiQol	**34**	65.52 ± 11.87	70.73 ± 13.84	−3.966	**<0.001**
Core FertiQol	**24**	68.14 ± 13.71	74.01 ± 15.65	−3.788	**<0.001**
Physical and psychological health	6	66.84 ± 18.92	74.47 ± 19.28	−3.858	**<0.001**
Emotion reaction	6	68.41 ± 17.68	75.31 ± 19.03	−3.650	**<0.001**
Social relations	6	69.61 ± 14.63	74.78 ± 16.43	−3.243	**0.001**
Marriage relations	6	67.68 ± 15.87	71.50 ± 17.44	−2.234	**0.026**
Treatment FertiQol	10	61.95 ± 14.21	65.52 ± 16.22	−2.293	**0.022**
Treat endure	4	66.72 ± 21.14	70.89 ± 21.56	−1.887	0.060
Treat environment	6	57.18 ± 14.00	60.15 ± 17.12	−1.876	0.061
Social support	**10**	37.32 ± 6.41	40.19 ± 7.51	−4.033	**<0.001**
Subjective support	4	21.67 ± 3.93	23.53 ± 4.69	−4.244	**﹤0.001**
Objective support	3	8.69 ± 2.98	9.32 ± 3.09	−2.001	**0.046**
SUPPORT utilization	3	6.97 ± 1.85	7.34 ± 2.17	−1.756	0.080
Fertility stress	**45**	169.29 ± 30.40	159.18 ± 29.71	3.231	**0.001**
Social stress	10	33.72 ± 8.35	31.36 ± 7.84	2.778	**0.006**
Sexual pressure	8	27.91 ± 6.84	24.97 ± 6.62	4.205	**<0.001**
Conjugal relations	10	37.94 ± 7.72	35.28 ± 8.07	3.271	**0.001**
Parental role needs	10	41.11 ± 8.29	39.13 ± 9.03	2.222	**0.027**
No child pressure	8	28.61 ± 7.52	28.45 ± 7.70	0.203	0.839
Mindfulness	**15**	63.44 ± 12.36	61.89 ± 13.66	1.155	0.249

Statistics with statistical differences are marked in bold for clarity; the total number of entries in the scale is marked in bold for clarity.

### Correlation analysis of the fertility quality of life, social support, fertility stress, and mindfulness

**Figure 1:**
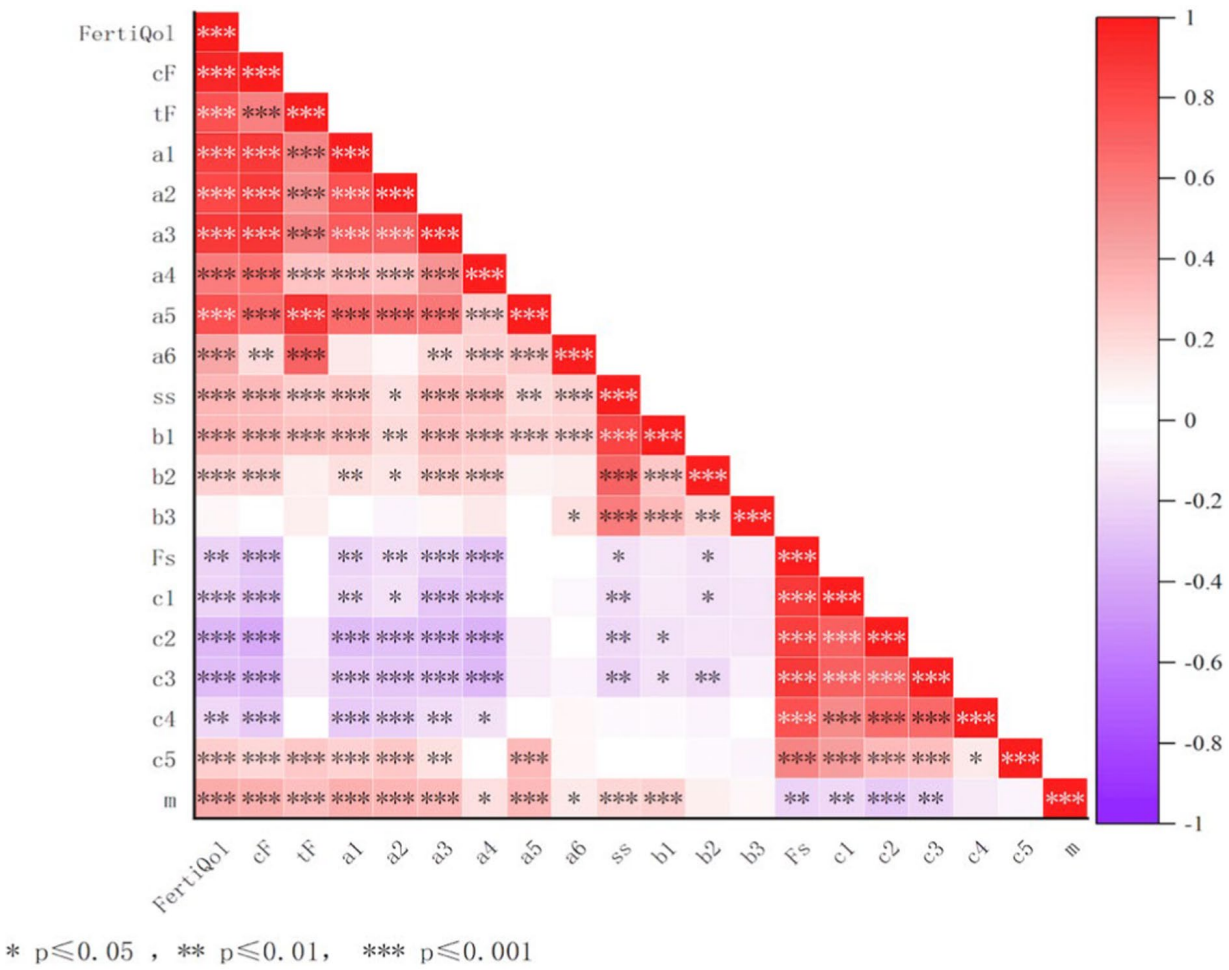
Correlation analysis among fertility quality of life, social support, fertility stress and mindfulness in infertile men. m, mindfulness; ss, social support; Fs, fertility stress; *F,* core FertiQol; tF, treatment FertiQol; a1, physical and psychological health; a2, emotion reaction; a3, social relations; a4, marriage relations; a5, treat endure; a6, treat environment; b1, subjective support; b2, objective support; b3, Support utilization; c1, social stress; c2, Sexual pressure; c3, conjugal relations; c4, Parental role needs; c5, No child pressure.

Figure 1 shows the results of the correlation analysis of the dimensions of fertility quality of life, social support, fertility stress, and the mindfulness for the case group. The total scores on the fertility quality of Life was positively correlated with the total score on the mindfulness and the scores on the social support (*p* < 0.001), whereas it was negatively correlated with the total and subscale scores of the fertility stress (*p* < 0.01).

### Dual mediating effects of mindfulness and social support in the relationship between fertility stress and fertility quality of life

**Figure 2:**
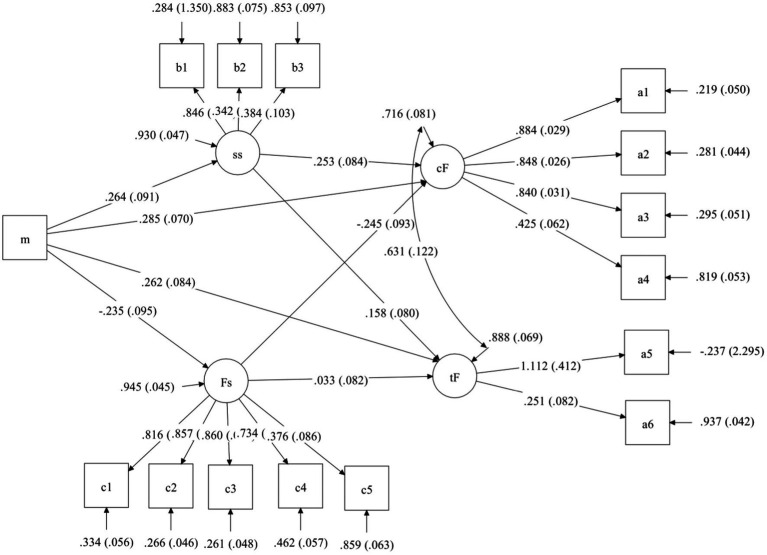
m, mindfulness; ss, social support; Fs, fertility stress; *cF*, core FertiQol; tF, treatment FertiQol; a1, physical and psychological health; a2, emotion reaction; a3, social relations; a4, marriage relations; a5, treat endure; a6, treat environment; b1, subjective support; b2, objective support; b3, Support utilization; c1, social stress; c2, Sexual pressure; c3, conjugal relations; c4, Parental role needs; c5, No child pressure.

The correlation between fertility stress, mindfulness, social support, and fertility quality of life reached a significant level. Based on the existing research, this study used the maximum likelihood method to establish a model and analyzed the mediation effect. Considering mindfulness as an external latent variable, social support (subjective support, objective support, and support utilization), fertility pressure (social pressure, sexual pressure, marital relationship, parental role demands, and childless pressure) as dual mediating variables, fertility life quality core modules (physical and mental health, emotional response, social relations, marital relations) and treatment modules (treatment tolerance, treatment environment) were used as internal latent variables to establish a mediating structure model. The results showed that the ratio of *χ*^2^ to degrees of freedom (*χ*^2^/df) = 3.35, CFI = 0.878, TLI = 0.843, RMSEA = 0.098 (*p* < 0.001), SRMR = 0.090, indicating that the fit of the structural equation model to the actual data was within the acceptable range. The mediation model diagram and path coefficients are shown in Figure 2. See Table 3 for legend.

**Table 3:** Legend of Figure 2.

Number	Name	Meaning	Graphics
1	Rectangular or square	Observed variables	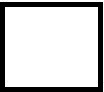
2	Oval or round	Latent variables	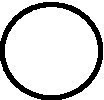
3	Single arrow	Unidirectional effects	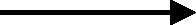
4	Double arrow	Correlation/covariance	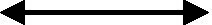
5	Single arrow pointing to ellipse	Latent variable residuals	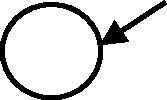
6	Single arrow pointing to rectangle	Measurement error	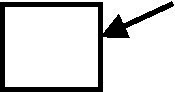

### The direct and indirect effect mediation test

Using repeated random sampling, 5,000 Bootstrap samples were drawn from the original data (*n* = 246), and 95% confidence intervals of the mediation effect were estimated using the 2.5th and the 97.5th percentile. If the 95% confidence interval for the indirect effect does not include 0, the mediating effect is significant. The results of this study showed that social support had a significant mediating role in the core and treatment modules of mindfulness and fertility quality of life, with confidence intervals of (0.014, 0.159) (0.004, 0.129), respectively. The mediating role of fertility stress in the core modules of mindfulness and fertility quality of life were significant, with a (0.012, 0.138) confidence interval, while the mediating effect of the treatment module was not significant, the confidence interval being (−0.071, 0.024). The mediating effect of social support on the core modules of mindfulness and fertility quality of life accounted for 19.0%, the mediating effect of mindfulness and fertility quality of life treatment modules accounted for 13.7%, and the mediating effect of fertility stress on the core modules of mindfulness and fertility quality of life accounted for 16.8% (Table 4).

**Table 4:** The direct and indirect effects.

Path	Normalized path coefficient	Standard error	95%*CI*
**Direct effect**			
Mindfulness → social support	0.264	0.091	(0.084,0.434)
Mindfulness → core FertiQol	0.285	0.070	(0.143,0.415)
Mindfulness → treatment FertiQol	0.262	0.084	(0.103,0.437)
Mindfulness → fertility stress	−0.235	0.095	(−0.411,﹣0.037)
Social support → core FertiQol	0.253	0.084	(0.094,0.431)
Social support → treatment FertiQol	0.158	0.080	(0.019,0.339)
Fertility stress → core FertiQol	−0.245	0.093	(−0.399,−0.040)
Fertility stress → treatment FertiQol	0.033	0.082	(−0.107,0.218)
**Indirect effect**			
Mindfulness → social support → core FertiQol	0.067	0.035	(0.014,0.159)
Mindfulness → social support → treatment FertiQol	0.042	0.029	(0.004,0.129)
Mindfulness → fertility stress → core FertiQol	0.058	0.030	(0.012,0.138)
Mindfulness → fertility stress → treatment FertiQol	−0.008	0.022	(−0.071,0.024)

## Discussion

Due to China’s particular cultural background, lineage inheritance is considered a major life event. Infertility not only causes considerable mental trauma to the patient himself but also causes immense pain to the two partners’ families. Relevant studies have confirmed that infertility causes male patients to experience considerable stigma and fertility stress (Jing et al., 2020). Most patients included in this study lived in cities, received college/undergraduate education, and had no children. The results of this study reported significant differences between the case and control groups in the core and treatment modules of fertility quality of life and significant differences in social support and fertility stress; however, the difference in mindfulness was not significant. Mindfulness in infertile men was significantly correlated with the core and treatment modules of fertility quality of life and indirectly affected these modules through social support, with the core module being affected by fertility stress.

There were significant differences between infertile and healthy men in terms of the total score of fertility quality of life, the scores of each dimension of the core module and the total score of the treatment module (*p* < 0.05), the quality of life of infertile men being lower than that of Sexty’s study of 234 infertile men in Germany (Sexty et al., 2018). Infertile men experience immense setbacks in physical and mental health, emotions, social relationships, and marital relationships, causing perceptions of social pressure and the development of inferiority complex, shame, and other emotions (Makara-Studzińska et al., 2022), leading to a decline in quality of life. Medical staff should attempt to create a harmonious and warm medical environment for male infertility patients. A good sperm retrieval and treatment environment is particularly important for these patients to face the issue more actively. A reduced quality of life can be caused by reduced social support due to infertility (Namdar et al., 2017). Due to various reasons (including “masculinity”), infertile men cannot truly face their own disease, and cannot share their physical and mental health problems with family members or medical staff. Consistent with the results of this study, infertile men are less socially supported than healthy men, receiving less love in terms of subjective and objective support, causing them to experience a sense of guilt about their family (Ried and Alfred, 2013; Keramat et al., 2014). The present results show that there are significant differences between infertile and healthy men in terms of fertility pressure, social pressure, sexual pressure, marital relationships, and childlessness pressure. The social pressure caused by infertility increases, while family and marital satisfaction decreases, with stress also being an important factor in reducing semen quality (Clemensb et al., 2014) and having adverse effects on infertility treatment (Rooney and Domar, 2018; Swift et al., 2021). Research shows that mindfulness helps control the body, which, in turn, controls the brain and reduces stress and anxiety (Esfahani et al., 2014; Jing et al., 2016; Family resilience, 2022). This is of great help to the core module of fertility quality of life, and it helps the continuity and tolerance of treatment for infertile patients. Therefore, mindfulness interventions for infertility patients can help relieve patients’ stress and improve their quality of life (Zhao et al., 2019), playing an important role in treatment effectiveness.

The results of the correlation analysis in this study showed that the fertility quality of life in infertile men was positively correlated with mindfulness and social support, and negatively correlated with fertility stress. According to previous findings, by establishing a structural equation model, the quality of fertility life can be divided into core and treatment modules, and an investigation the pathway from mindfulness to these modules has shown that mindfulness can directly affect fertility in infertile men (Li J. et al., 2019). Quality of life can indirectly affect the core and treatment modules of fertility life quality through social support, with a significant mediating effect, and can also indirectly affect the core module of fertility life quality through fertility stress, with a significant mediating effect. The core module of improving the fertility quality of life requires improving the level of mindfulness of patients. Mindfulness, as a protective factor, can play an important role in improving specific aspects of the fertility quality of life of infertile men (Li J. et al., 2019). The causal relationship path map of this study shows that mindfulness has the following five pathways affecting fertility life quality: mindfulness → fertility life quality core module (*β*= 0.285); mindfulness → social support → fertility life quality core module (*β* = 0.067); mindfulness → fertility life quality treatment module (*β* = 0.262); mindfulness → social support → fertility life quality treatment module (*β* = 0.042), indicating that the indirect effect of mindfulness is weakened after being mediated by social support; and mindfulness → fertility stress → fertility quality of life core module (*β* = 0.058), indicating that the indirect effect of mindfulness is weakened after being mediated by fertility stress. Consistent with previous research findings (Li et al., 2020), patients with a high level of mindfulness can better regulate their emotions to relieve the stress brought about by childbirth, which helps to eliminate strong stress and improve their quality of life. The shortcomings of the study are that the causal relationships between variables cannot be determined due to the limitations of the cross-sectional study, and there are confounding factors in the variables due to the limitation of the sample size. Therefore, needing to increase the sample size and make efforts to further enrich the research results. Meanwhile，In this study, social support had a positive effect on the core and treatment modules of fertility quality of life. The results of numerous studies (Schaller et al., 2016; Namdar et al., 2017; Maroufizadeh et al., 2018; Bhamani et al., 2020) have shown that social support is an important factor in the quality of life of male infertility patients. With full support from family, society, hospital, and partner, male infertility patients will be more positive in coping with the treatment process and overcoming their internal pain. Male infertility patients are reluctant to talk about their infertility status with their partners, family members, and colleagues in their social network, and even more reluctant to talk about their feelings and worries in the face of healthcare workers (Warchol-Biedermann, 2021). Structural equation modeling results show that fertility stress negatively affects the core modules of fertility quality of life. Compared with studies by foreign scholars (Moura-Ramos et al., 2012), fertility-related stress in male infertility patients in China was significantly higher than abroad, suggesting that male infertility patients in China are under greater stress and that domestic medical institutions tend to focus on treatment at the expense of psychological support for infertile patients, which may also account for the greater fertility stress in infertile men in China than abroad. The limitation of this study is that only the fertility quality of life of infertile men was investigated, and their spouses were not investigated at the same time, ignoring the interactions and differences between the fertility quality of life of spouses and infertile men.

## Recommendations

In this study, it was found that improving the level of mindfulness in infertile men could have a significant impact on the fertility quality of life indirectly through social support and fertility stress. Mindfulness interventions and other positive psychological interventions can therefore be used to prevent negative emotions in men with infertility from affecting their fertility quality of life and subsequent infertility treatment. Today’s shift in the biopsychosocial model of medicine has seen a greater focus on mental health in the development of disease. Interviews with specialists in reproductive medicine have revealed that the current frequency of infertility is related to pathological factors, but also more to men’s fear of gender life, their inability to enjoy and complete it, and the increasing number of physical factors of infertility. Therefore, healthcare professionals, family members, and patients with fertility problems can have some degree of psychological intervention to increase confidence and improve the fertility quality of life through online media and psychological counseling.

## Conclusion

This study analyzed the correlation between social support, fertility stress, mindfulness, and fertility quality of life in infertile men and explored the dual mediating effects of social support and fertility stress on the latter two. The conclusions were as follows: (i) Infertile men had lower fertility quality of life and social support and higher fertility stress and positive thinking compared to healthy men. (ii) There is a significant correlation between mindfulness and fertility quality of life, which can affect fertility quality of life not only directly but also indirectly through social support and fertility stress. An intervention program is offered to improve the fertility quality of life of infertile men. (iii) Social support positively influences the core and treatment modules of fertility quality of life, and fertility stress negatively influences the core modules of fertility quality of life.

## Data availability statement

The original contributions presented in the study are included in the article/supplementary material, further inquiries can be directed to the corresponding authors.

## Ethics statement

The studies involving human participants were reviewed and approved by Ethics Review Committee of the First Affiliated Hospital of Xinjiang Medical University (20210226-168). The patients/participants provided their written informed consent to participate in this study.

## Author contributions

MA contributed fully to the study, including study setup, data processing, manuscript writing, analysis review, manuscript drafting, and review. HX contributed to the discussion section and reviewed the paper. AA contributed to the data analysis and paper review. WZ contributed to the data collection and paper review. LH contributed to the methods section and reviewed and revised the paper. CZ contributed to the introduction section and reviewed the paper. All authors contributed to the article and approved the submitted version.

## Funding

This work was supported by Xinjiang Key Laboratory of Special Environment and Health Research Open Subjects (SKL-SEHR-2021-05), National Natural Science Foundation (82260651) and Post-doctoral Research Start-up Grant.

## Conflict of interest

The authors declare that the research was conducted in the absence of any commercial or financial relationships that could be construed as a potential conflict of interest.

## Publisher’s note

All claims expressed in this article are solely those of the authors and do not necessarily represent those of their affiliated organizations, or those of the publisher, the editors and the reviewers. Any product that may be evaluated in this article, or claim that may be made by its manufacturer, is not guaranteed or endorsed by the publisher.
